# The Silk Worm Epidemic, Pebrine, after Pasteur, with Some Reflections Regarding the Analogy of This Disorder to Certain Diseases of the Human Subject

**Published:** 1877-02

**Authors:** John Bartlett


### THE SILK WORM EPIDEMIC, PEBRINE, AFTER PASTEUR, WITH SOME REFLECTIONS REGARDING THE ANALOGY OF THIS DISORDER TO CERTAIN DISEASES OF THE HUMAN SUBJECT.

By JOHN BARTLETT, M.D.

(A Paper read before the Chicago Medical Society, Nov. 20, 1876.)*

In 1849 the silk crop of France, then estimated at 325,000,000 lbs., in a great measure failed, because of a disease, the pebrine, epidemic among the silk worms. This failure, growing year by year more serious, induced the French government in 1865 to assign the task of determining the nature of, and remedy for, pebrine, to the distinguished investigator Pasteur. In 1870 appeared the remarkable monograph of this scientist entitled "Etudes sur la Maladie des Vers à Soie," a synopsis of which forms the basis of this paper.

The pebrine is a contagious, infectious and hereditary disease, the cause being the introduction into the system of the worm of a psorospermoid cell, and the growth and development of this in the larva, pupa, moth or egg, leading finally to the destruction of the organism upon which it preys. The name *pebrine*, signifying pepper, has reference to one of the most palpable symptoms of the disease — the discoloration of the skin by numerous black spots or points, suggestive of the insect's having been dusted with pepper.

The *symptoms* as occuring in the worm, are loss of appetite and vigor, and failure of nutrition, as observed in stunted growth. The spots appear primarily on the head and neck as very minute specks ; at first yellow, then brown and black. Meanwhile the primitive yellow discoloration invades the neighboring tissues as an areola of the dark spot. Sometimes death results before the period of cocooning arrives; in other cases an imperfect cocoon is formed, or the insect, day after day, makes the to and fro motion of the head, neck and body, as in the act of spinning, no silk meanwhile issuing from the

* Condensed by the Author for this Journal.

silk duct, and the worm finally dying without a covering. The cocoons of pebrined worms are feeble in silk, and this is of a poor quality. The chrysalis may die, or if the disease be mild, it may develop a moth, which may or may not be able to pierce the cocoon and emerge. The moth, if only slightly affected, may present no evidences of disease in appearance, action or function, or such evidence may be manifest in sluggishness of movement, inability to copulate and to lay. When the disease in the miller is severe, it is revealed by striking symptoms; one of these is a dark gangrenous appearance of the posterior half of the body, the other a shrivelled œdematous and ecchymosed condition of the wings. The eggs of badly infected caterpillers are sometimes sterile; if they hatch, worms are produced which speedily perish of pebrine of a ten fold more virulent form than that which affected their ascendants. As the disease is eminently contagious, hereditary and fatal, the havoc made by it would soon prove annihilating in any districts not supplied with healthy stock from abroad.

With the *cause* of the disease, Pasteur associated a brilliant ovoid corpuscle, first noticed in the bodies of pebrined worms by Cornalia, but regarded by him as accidental. Most careful and varied experiments conducted by Pasteur demonstrated that there was no pebrine without this corpuscle, and no corpuscle without pebrine, and that the disease could be produced at will by introducing these germ cells into the bodies of healthy worms. In infected broods, the corpuscles were found in great abundance in all the tissues and fluids of the worms, chrysalides and moths. They existed in the eggs, and were found in large numbers in the litters, and in the dust of the worm houses.

*Morbid Anatomy.* The intestinal walls were infiltrated with corpuscles; in the skin, in the cellular tissue underlying the pebrined spots, they existed as masses. The silk glands were densely packed with them, so that a section presented a porcelained appearance. This occupation of the glands by the germ cells led at first to the exclusion of their proper secretion and finally to the complete destruction of the organ. The same was true of the malpighian tubes. The blood of the insect teemed with corpuscles—in short, the aggregation of the disease-cell in all the tissues of the worm ceased only with the destructive occupation of them. In the pupa and moth, substantially the same appearances were presented; in all the tissues a replacement of proper structure by the formed material of the disease-cell had to a great extent occurred. In the egg the corpuscles could be detected; and if numerous, the integrity of the ovum was destroyed.

*The Cell.* Upon proceeding to the study of the corpuscle to which reference has been made, Pasteur could at first discover no way in which the multiplication of it was accomplished. Ultimately he learned that this corpuscle seen by Cornalia, and so conspicuous in worms dead of pebrine, was inactive, effete. It was not the living cell occasioning the disease, but only the formed material of such cell. That it was an effete organism incapaple of reproduction, or of inoculating pebrine, was fully established by unsuccessful attempts to inoculate worms with the old dust of infected breeding houses, with the debris of worms one year dead, and with corpuscles washed from the surface of eggs known to be corpuscular.

Pasteur subsequently discovered the young cellule from which through varied changes the adult corpuscle sprang. These cellules when just detectable seemed to have two forms, both very small, pale and indistinct, at first homogeneous and finally exhibiting vacuoles, and contained nucleated granules, which, after splitting into lesser segments developed into corpuscles. In their long diameters, the disease-cells measured one ten-thousandth of an inch.

Of the natural history of the noxious corpuscle, its nidus or habitat, nothing more is known than that it was first observed by Leydig in 1853, in the fluids and tissues of the cochineal insect, of spiders and crawfish. Where it more naturally belongs, and what causes may lead to its more frequent appearance in silk worms, are alike unknown.

*The development and growth of the corpuscle in the worm* was studied by poisoning a brood by sprinkling a mixture obtained by triturating a corpuscular moth in a mortar with a little water.. On the fifth or sixth day of the poisoning, cellules began to appear in the intestinal walls, remaining isolated therein for five or six days and finally aggregating into masses in all of the organs. The ingested corpuscles soon manifested their effects on the skin. Two or three days after the appearance of the cellules in the walls of the intestines, and before they were elsewhere discoverable, spots appeared on the integument, at first very minute, but becoming quite distinct on the tenth or twelfth day of infection. The appearance of these spots prior to the general invasion of the skin, was considered by Pasteur as remarkable.

The disease is *inoculable.* By mixing corpuscular worm tissue with a little water, and inoculating sound insects with the virus, poisoning results. Accidental inoculation is one of the modes in which the disease is conveyed. Pasteur noted on the bodies of worms very minute wounds, some of these were incised, others were more in the form of ulcers resembling finally the true pebrine spot. Upon careful observation he noted that the simple wounds so generally seen on the skin were inflicted by the claws of the insects as they crowded one over the other in their baskets. The spots having the appearance of ulcerations, were inoculated incisions. The hooks with which the fore feet of the insect are provided, have on their inferior surface a concavity. Washing out these minute receptacles for dirt, and placing the contents under the microscope, pebrine cells were detected. Numerous facts demonstrate the disease to be *infectious.* Thus, if in a contaminated worm house an education of healthy insects be placed under ordinary circumstances, it will be infected sooner or later. The disease in such instances may be carried from the sick to the healthy by the person of the attendant, or the cell may be conveyed by the air. For the most part under the circumstances of exposure just cited, Pasteur is of the opinion that the disease is conveyed by the contact of attendants. For experiment shows that a healthy education may be reared near an unhealthy one and remain pure, or nearly so, provided that the greatest pains be taken that no direct convection of contagious particles shall take place between the infected and the well.

It would seem that the distances to which the disease may be carried by the air from a contaminated education is less than might be supposed, it being practically possible to preserve worms for industrial purpose free enough from pebrine, when separated from moderate foci of infection 30 yards. Pasteur thinks the instances must be rare in which the disease is extensively communicated by leaves holding on their surfaces the pebrine corpuscles. Cases in which such a source of contagion has been thought to exist, could more naturally be referred to convection through human intercourse. And yet, as a single corpuscle is sufficient to ingraft the disease, Pasteur is of the opinion that a distance of 30 miles should, when possible, be placed between badly infected districts and worm houses which it is proposed to keep pure.

The *heredity* of the disease is as well established as its contagion and infection. Numerous experiments have demonstrated that in the very great majority of cases, the eggs, the progenitor of which, whether in the larva, chrysalis or butterfly state, contained corpuscles, would give issue to infected worms. Sometimes a moth, containing comparatively few corpuscles, may have begotten eggs, some or all of which are free from corpuscles. In such a chrysalis and miller, the tissues of which are but sparsely infused with corpuscles, it may be supposed that by chance no pebrine cell had found its nidus in the ovary. In the eggs of unhealthy worms, corpuscles, in all stages of growth, were found in great numbers. Sometimes, however, in the ovum, as in other tissues, in the earliest periods of the existence of the pebrine cells, they were too small and pale to be visible, and it was in some instances only after considerable time had elapsed, sufficient to allow of the growth of the cells to visibility, that they could be detected. Pasteur inclined to believe that the diseased cell was inherited from the female only. Copulation of a healthy female with an infected male did not, in his limited experiments, result in infection of the eggs, and yet, feeling that his observations were too few to decide the question of transmission through the male, he recommends, in his process for procuring perfect grain, that lots impregnated by very corpuscular males should be avoided.

Numerous carefully conducted experiments demonstrated that the cellule, at least when exposed for some time to temperature, moisture, etc., lost its vitality. Thus it was impossible to inoculate pebrine from corpusclar matter taken from the infected worms or moths of the previous season, or from the last year's dust of the breeding houses. In fact, an experiment showed that inoculation was unsuccessful when performed with germs from a corpuscular worm which had died six weeks before, and had dried in the air. On the contrary, infection was successfully effected when the virus was obtained from worms dead a few weeks, and not mummified.

Enclosed in the protecting shell of the egg, cellules preserve their infecting power from year to year, and there is reason to believe that germs encased within the crust of the chrysalis have retained their potency as long as four years. It has been stated that during the lethargic condition of the worm at the moultings it was feeble and especially liable to disease. During these periods the development and growth of the germ-cells were noticed to be particularly active. A high temperature seemed also to favor their increase.

It will be well here to comment on two erroneous impressions of many sericulturists; the one that the pebrine is not contagious, the other that it is local, pertaining to certain districts, as infected centers. The gentle, gradual advance of the malady, so that an infected worm may yet form its chrysalis, and this its moth, and this beget its progeny before the disease breaks out in virulence, has led observers, overlooking the steps by which the real cause of the infection has advanced, to regard the actual outbreak of the disorder as an epizooty, as the initial period of the attack. And in the absence of any apparent source of immediate contagion the existence of infection is denied, and the conclusion is reached that the disease can only be referred to an all pervading miasm.

It has been stated that worm tissue containing cellules exposed to atmospheric changes for as long a period as six weeks, lost its power of infecting with pebrine. It is a remarkable fact, however, that it is still because of the contained mature corpuscle, highly poisonous, and if it be introduced into the alimentary canal of worms, however old it may have become, it gives rise to a disease fatal within two or three days. This disease, long known to silk growers as *flacherie*, appears to be due to the development of vibrios in the intestinal canal, begetting fermentation in the ingested food, and destroying the worm by inanition.

The *diagnosis* of pebrine is easy and certain, the only essential evidence of the existence of the disease is the presence of its corpuscle; cells very young and in the nascent state are probably invisible to the highest powers. At the very early period of the disease, before the germ cells are well developed, their existence might be overlooked; it is desirable therefore to allow time for their growth before a diagnosis is made. Thus, in the newly formed chrysalis, of the very slightly infected worm, no corpuscles may be found; but in one week or a fortnight they may be plainly seen. And in the freshly laid eggs no signs of disease may be detected, but in a few weeks or months the grain may be found to be very corpuscular. The adult corpuscle, though not the cause of disease, is the sign of the probable presence of that cause; it may therefore be depended upon as a means of diagnosis.

Treatment.—Of the various remedies tried for the prevention or cure of the pebrine, no one was in any way efficacious. These facts only are worthy of record: Water saturated with carbolic acid kills the germs, but fumigation with this acid was worse than useless. Chlorine fumigations prove destructive to the cells; but it is not stated whether the presence of chlorine in sufficient quantity for this result is compatible with the life of the worm itself.

*Hygiene.* The hygiene of this disease is as satisfactory as the treatment is discouraging. It has reference to the surroundings of the worms, and more paticularly to their parentage.

It is of the first importance that the parentage should be perfect, or nearly so. Before the investigations of Pasteur it was the practice of silk raisers to select the best appearing cocoons from which to obtain the butterfly for the deposit of the eggs, as the seed for the next harvest. Pasteur recommended that a large number of female moths, say 50 or 100, taken from lots which it is desired to use for grain, should be carefully examined, and if they be free from corpuscles, the eggs produced by broods from which they have been selected may be used with every confidence of a successful result. His plan is to take one or two millers at a time, of those to be tested, and triturate them in a mortar containing a few drops of water. Portions of the resulting mass are subjected to the microscope and the presence or absence of corpuscles determined. When it is desirable to be very particular, both the female and male progenitors of the eggs in question are submitted to examination in the manner stated, and if a single corpuscle be discovered their eggs are rejected. On the contrary, if the scarcity of seed grain is great, or no millers perfectly free from corpuscles can be had, the product of butterflies, ten per cent. of which are moderately corpuscular, may be used for reproduction. For experience has shown that when the infection of the ascendants is not marked it is possible for the eggs to produce worms sufficiently healthy for industrial purposes, because, in cases of slight infection of the parents, such is the slowness of development of the corpuscle, that a perfect cocoon may be formed before the destructive power of the cell has accumulated.

Having secured eggs free from germs of disease, it is essential to preserve the worms hatched therefrom from all infecting influences. The corpuscles, we have seen, may be conveyed to sound worms by the air from neighboring breeding houses, or by the attendant upon infected broods. The healthy lot then should be kept at a distance from the infected one, and there should be no communication by person or utensil between them.

As the corpuscles abound in the dejections of the worms, these should be frequently removed and destroyed, and care should be taken especially that the leaves fed to the worms be not soiled by the evacuations. Over crowding should be avoided for obvious reasons, namely: the greater the multitude of worms the greater is the chance of the presence of corpuscles, and the greater is the probability that the diseased germs may in some way be received in some one member of the same basket or worm house by ingestion or inoculation. Inasmuch as the corpuscles of the preceding year, resting as dust on the tables, floors, etc., of the breeding rooms, though inert as to pebrine, are yet poisonous, it is essential that in the beginning of the season the houses should be thoroughly cleansed and disinfected, and that thereafter dust should be removed regularly, and that by a mopping rather than a sweeping process.

REMARKS.—The pebrine in its symptoms and termination finds no exact analogue in disorders of man, and yet its resemblance to several diseases would occur to all. In its fatality, heredity, and in its capability of inoculation, as well as in the manner in which it invades the tissues, as the silk glands, it is suggestive of phthisis. Such a comparison implies that the gray granulations of Bayle are masses of cells, and formed material of cells.

In its inoculability, its gravity, its transmissibility from mother to offspring, as also in its deposits and surface manifestations, it resembles syphilis.

The plague, with its contagion, infection and deadly character, with its adventitious deposits, its carbuncles, gangrenous patches, petechial ecchymoses and serous effusions, would appear much to resemble the devasting pebrine of France.

In several of its features the history of pebrine suggests the possibility that the poison of small pox, typhoid fever and glanders may be found to depend upon the invasion of the human tissues by a cell growth heterologous and parasitic.

In regard to tubercle, while its main forms are but an aggregation of corpuscles, or as Dr. Fox says, "a growth of densely massed small cells," even more like those of pebrine than the growths of syphilis, yet, in view of the recent pathology which tends to remove from the tubercle all specific characters, and to class its products with those of inflammation of lymphatic tissue, some may deem the comparison of it to a disease produced by the growth of a foreign cell in the tissues hardly allowable. But I need hardly remind gentlemen who have read the discussion on tubercle before the Pathological Society of London a few years since, by such men as Wilson Fox, Beale, Bastian, and their colleagues, that the question of the nature of tubercle is yet *sub judice.* Allow me here to cite some expressions of Beale in the discussion referred to. He said: "I cannot think that tubercle corpuscle has anything to do with the lymph corpuscle or with the cells of adenoid tissue. Nor do I understand how any process that may be denominated irritation can convert one of these lymphatic corpuscles into tubercle. It seems to me that tubercle consists of living matter, having the power of multiplication, and differing greatly from any cell in the body. The tubercle corpuscle is a special thing."

In his book on disease germs, Dr. Beale writes: "There is reason to think that particles of living, growing tubercle exist sufficiently minute to be supported by the atmosphere, and carried long distances. There are many facts, considered by some sufficiently conclusive, to justify the opinion that tubercular disease of the lung is, at least in some instances, contagious. If a particle of fluid holding living tubercle germs in suspension were introduced by inoculation into a weakly organism, the disease might be produced. From collections of tubercles bioplasts may be readily detached, and might be carried to distant parts and grow there." I may add in connection with this comparison of pebrine and phthisis two interesting statements as to the propagation of the latter disease. Læennec states that having lacerated his hand with a saw while examining a body dead of phthisis, an inoculation followed which resulted in the growth of a tubercular nodule at the place of injury, which was enucleated. Dr. Jacobi reports that his dog, having been allowed to eat the sputa of a phthisical patient, died presently of consumption. I desire here to suggest that the true tubercle cell may first exist, like the pebrine cell, as a nascent, almost or quite invisible, cellule, then as a matured cell, and finally, having undergone tyrosis, as a yellow tubercle. The supposition that tubercle cellules may exist and grow at the expense and to the injury of the lung, without appearing as a collection of mature formed material corpuscles, would explain the view insisted on by Niemeyer, that patients die of phthisis in whose lungs no tubercles are discoverable.

In this connection it is interesting to bear in mind the following statement of Dr. Crisp: "He had met with tubercle in more than one hundred different species of animals, quadrupeds, birds and reptiles. In 1854 there was a great mortality among the sparrows of Regent's Park gardens. He examined many of these, and found the livers, spleens and intestines tuberculous; these birds fed with a large number of tuberculous animals. Reptiles in confinement are very subject to tubercle in the liver, intestines, spleens and lungs; large masses often block up the alimentary canal. He had observed four stages of the disease: the first the hyperæmic, congestive or inflammatory (query, the stage of growth of cellules?); secondly, the deposit of cell growth; the third that of softening; fourth, that of cretaceous change. Vegetable feeders, as birds, are more liable to tubercle. It is hereditary, and he believed, in the lower animals contagious."

The resemblance of pebrine in its pathological histology to syphilis is quite close. The induration of chancre and the gummata of syphilis are but infiltrations of connective tissue by small cells, not dissimilar to those of pebrine, and these cells readily admitted to be heterologous and specific.

The phenomena of contagion and infection, as observed in the silk worm epidemic, are so similar to those of diseases in higher animals that it is hardly necessary to comment upon them. Two facts, however, invite our attention: one, that while under exposure the germ cell loses its vitality; under other conditions of protection it retains it for years, and possibly indefinitely. The other fact, as remarkable as suggestive, is that the mature old cells of pebrine, while they are incapable when inoculated or ingested of reproducing the original disorder, yet beget another most destructive, contagious and infectious distemper. Can it be possible that in the human subject a disease as the scarlet fever, or chancre, may have for its cause a germ, which when modified by age or other circumstance, may produce another form of illness, as the diphtheria or chancroid? Yet another item of interest may be found in the discovery of Pasteur, that silk worm moths preserved in museums since 1838 contained pebrine corpuscles, not to be distinguished by the sight from recent ones. What possibilities in the way of storing up of germs of contagion does this not suggest! As the living seed of grain may be preserved in the mummies of Egypt for thousands of years, so may it be possible that suitable containers may serve, like the box of Pandora, to transmit distempers from generation to generation.

The detection of the cell germ in the egg as the determining cause of disease in the descendant, is explanatory of the mode of transmission of ailments from parent to offspring. The fact that some moths not very corpuscular beget healthy worms is also of interest, as confirming the observation that a syphilitic mother *may* give birth to a healthy child. According to Pasteur's observations, when ten per cent. of the moths of a given lot of worms were corpuscular, not more than two per cent. of the eggs of this lot would prove to have been contaminated.

I call attention to the excellent illustration of incubation which the history of the pebrine cell furnishes. Very slowly the germ of disease increases to such numbers as to interfere with the functions of the insect. It is noteworthy also that the rate at which this increase occurs is affected by temperature, and by certain favoring states of the worm. Multiplication being more rapid at high temperatures, and during those periods of the life of the insect when it is most feeble.

The phenomenon of latency is also admirably illustrated. Incased in the shell of the egg, and probably also in the sheath of the chrysalis, the corpuscle may lie dormant in the tissues, till an event, as the full development of the larva or pupa, excites it into activity.

The cell germ is regarded by all who have studied it as vegetable. We see virus from one animal inducing disease, as vaccinia, when inoculated into another. In this instance the contagioning atom is probably an animal structure. Leydig has discovered in certain insects, other than the silk worm, cells apparently identical with the pebrine corpuscle. These, the discoverer believes, belong to the normal cell-structure of those insects. Now may it not be possible that the corpuscle so noxious to the Bombyx Mori may be a healthy cell of the spider, cochineal insect, or crawfish? In other words, may not a cellule normal to one organization prove the source of disease to an animal of a different class, by preying as a parasite upon its tissues? And may not such a possibility serve to explain the production in one animal of a tuberculoid disease simply by the inoculation of the products of inflammation from another animal?

I desire particularly to invite your notice to the statement of Pasteur, that in the very early origin of the cell they are invisible, and when first seen they are very pale, indefinite in outline, and barely distinguishable. Leydig confirms this observation, and it is exactly in accordance with my own experience in the study of the extremely minute atom associated, as I believe, with ague. We may adduce from this fact a lesson in reference to *post-mortem* diagnosis, namely, that tissues may be invaded with germ cells of disease in such numbers as probably to destroy life, and yet be invisible to very high powers of the microscope. In view of such a possibility, it is wise to subject specimens to examination at different intervals of time, so as to allow opportunity for the growth of any infinitely minute disease cells to visibility. And finally, an abnormal cell or atom discovered in a tissue is not to be regarded as having been always inert, because experiment shows it to be, in the state in which it was found, incompetent to produce disease. For the cell so experimented with may, like the mature pebrine cells, be no longer capable of reproduction, and in the phase of life in which it was found prove entirely inactive; whereas, at an earlier period, it may have been capable of the most destructive mischief. In short, the pathologist must not mistake formed material for the living cell, lest, finding the former inert, he may erroneously deny potency to the latter.

The physician will do well to note that the efficiency of measures taken by Pasteur to prevent infection, namely, the removal of dead worms, the clearing away and destruction of the dejections. The avoidance of too close proximity to diseased localities, and the prevention of indirect communication between the sick and well, cannot but add some corroboration to the germ theory of such diseases as the cholera, for the prevention of which experience has taught the wisdom of exactly similar hygienic rules.

Observe also the direction to protect the food of the worm from the contagioning atom. The fact of the entrance of the multiplying pebrine cell through the alimentary canal may tend to deepen the growing impression that milk may be a vehicle of contagion.

A sarcastic reference of Cornalia to the "copious and useless pharmacopœia of the silk worm as extensive as that of man," cannot but remind the practitioner of the random application of means so often made to diseases of the human subject, the causes of which are probably as little within the reach of such medication as was the cell of pebrine to the multiform remedies vainly prescribed against it.

